# Advanced predictive modeling for enhanced mortality prediction in ICU stroke patients using clinical data

**DOI:** 10.1371/journal.pone.0323441

**Published:** 2025-05-28

**Authors:** Armin Abdollahi, Negin Ashrafi, Xinghong Ma, Jiahao Zhang, Daijia Wu, Tongshou Wu, Zizheng Ye, Maryam Pishgar

**Affiliations:** Andrew and Erna Viterbi School of Engineering, University of Southern California (USC), Los Angeles, CA, United States; Hamad Medical Corporation, QATAR

## Abstract

**Background** Stroke is second-leading cause of disability and death among adults. Approximately 17 million people suffer from a stroke annually, with about 85% being ischemic strokes. Predicting mortality of ischemic stroke patients in intensive care unit (ICU) is crucial for optimizing treatment strategies, allocating resources, and improving survival rates.

**Methods** We acquired data on ICU ischemic stroke patients from MIMIC-IV database, including diagnoses, vital signs, laboratory tests, medications, procedures, treatments, and clinical notes. Stroke patients were randomly divided into training (70%, n=2441), test (15%, n=523), and validation (15%, n=523) sets. To address data imbalances, we applied Synthetic Minority Over-sampling Technique (SMOTE). We selected 30 features for model development, significantly reducing feature number from 1095 used in the best study. We developed a deep learning model to assess mortality risk and implemented several baseline machine learning models for comparison.

**Results** XGB-DL model, combining XGBoost for feature selection and deep learning, effectively minimized false positives. Model’s AUROC improved from 0.865 (95% CI: 0.821 - 0.905) on first day to 0.903 (95% CI: 0.868 - 0.936) by fourth day using data from 3,646 ICU mortality patients in the MIMIC-IV database with 0.945 AUROC (95% CI: 0.944-0.947) during training. Although other ML models also performed well in terms of AUROC, we chose Deep Learning for its higher specificity.

**Conclusion** Through enhanced feature selection and data cleaning, proposed model demonstrates a 13% AUROC improvement compared to existing models while reducing feature number from 1095 in previous studies to 30.

## Background

The intensive care unit (ICU) is a structured system designed to care for critically ill patients, offering intensive and specialized medical and nursing services, advanced monitoring capabilities, and multiple physiological organ support modalities to sustain life during periods of severe organ system failure [1]. In the United States, stroke is a leading cause of death and disability, underscoring the critical importance of ICU care for stroke patients [2].

Ischemic stroke occurs when blood flow to the brain is blocked or reduced, posing significant health risks [3]. In recent years, approximately 13.7 million people suffer strokes annually, with 5.8 million resulting in death, of which 70% are ischemic strokes [4]. The large number of stroke patients significantly exacerbates the challenge of proper ICU resource allocation, particularly during the COVID-19 era. Logistically, there is a severe shortage of equipment and medications (such as ventilators and syringe pumps), while the number of patients far exceeds hospital capacity, preventing medical staff from providing timely treatment [5]. Stroke patients requiring intensive care are at extremely high risk of short-term death, although this risk diminishes with increased survival time following ICU admission [6].

ICUs also cater to patients with other critical conditions. For instance, machine learning models have been developed to predict in-hospital mortality for ICU patients with heart failure, demonstrating the utility of advanced algorithms in critical care settings [7]. Similarly, deep learning models have been utilized to predict mortality in mechanically ventilated ICU patients, highlighting the significance of predictive analytics in managing complex ICU cases [8].

From a genetic standpoint, hereditary conditions such as hypertension and diabetes may be passed down through familial bloodlines, increasing the potential risk of stroke in otherwise healthy individuals [9–11]. Alternatively, harmful lifestyle practices, such as smoking and lack of exercise, are also significant factors leading to the frequent occurrence of strokes [12].

With the advent of machine learning, algorithms have been increasingly applied to various disease prediction models [13, 14]. Compared to traditional statistical methods, machine learning can rapidly process numerous features, consider more permutations, and enhance prediction accuracy [15, 16]. A substantial proportion of machine learning models developed for disease analysis focus on stroke patients [17, 18]. These mortality prediction models for stroke patients are widely used in clinical medicine to provide timely warnings to ICU doctors and to facilitate the efficient allocation of medical resources [19, 20].

Neural network models and deep learning represent the forefront of artificial intelligence, transforming how machines process information and make decisions [21–23]. Neural networks mimic the interconnected neurons in the brain to process complex data, and one of their key strengths is the ability to learn intricate patterns and relationships from data without explicit programming [24, 25].

The primary objective of this research was to develop a deep learning model for predicting the mortality of ischemic stroke patients using ICU patient data from the MIMIC-IV database. Compared to the primary reference article [20], we employed feature selection to reduce the number of predictor variables while improving the accuracy of the results. The predictive model was developed following the guidelines of the Transparent Reporting of a Multivariable Prediction Model for Individual Prognosis or Diagnosis (TRIPOD) initiative [26].

## Methodology

**Fig 1:**
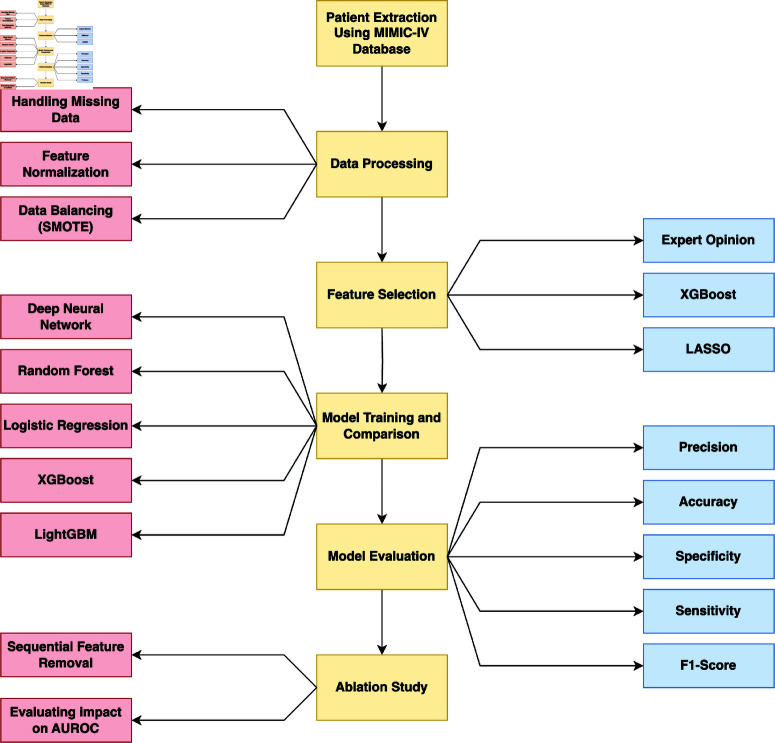
Study workflow. Diagram of the methodologies adopted in this study.

In this study, we aimed to develop a robust predictive model for ICU stroke patients’ mortality using the MIMIC-IV database. The overall methodology consists of several key steps, which include patient extraction from MIMIC-IV, Data processing, feature selection, model training, and ablation study. These steps were crucial in building and refining our model to ensure high performance and reliability. The methodology is summarized in Fig 1, which provides a comprehensive overview of the entire process, from data preprocessing to model evaluation and comparison across different machine learning techniques.

## Data source and study design

Our study utilized the Medical Information Mart for Intensive Care (MIMIC-IV) database, a contemporary electronic health record dataset resulting from a collaboration between Beth Israel Deaconess Medical Center (BIDMC) and the Massachusetts Institute of Technology (MIT) [27]. Specific data, including patient diagnoses, vital signs, laboratory tests, medications, procedures, treatments, and de-identified free-text clinical notes, were extracted from the MIMIC-IV database to cover specific patient cohorts. The MIMIC-IV database was chosen because it provides an extensive amount of real ICU patient data and, compared to MIMIC-III, offers more accurate updates and organizes the data into a modular structure. This facilitates the formulation of hypotheses for more comprehensive research problems and their application in clinical medicine. After data extraction, preprocessing is essential to ensure high data quality and to organize it into a format suitable for analysis by machine learning algorithms. The data within the MIMIC-IV database serves as a robust foundation for research endeavors, effectively supporting the development of deep learning models and benefiting clinical medical personnel.

## Patient extraction

**Fig 2:**
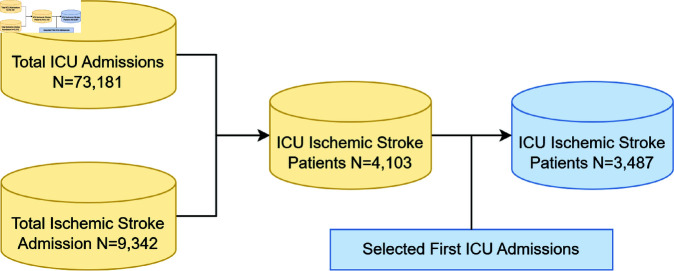
Patient selection. Flow diagram of the selection process of patients.

Our research focused on predicting mortality in ICU patients with ischemic stroke. Fig 2 illustrates the patient extraction process. Initially, we selected 73,181 ICU patients and 9,342 ischemic stroke patients from the database. From these, we identified 4,103 patients with ischemic stroke in the ICU. Ultimately, we included the first ICU admission for each patient, resulting in a total of 3,487 patients who met the established inclusion criteria for the final analysis.

We exclusively included first-time ICU admissions for each patient in this study to maintain the consistency and reliability of the dataset. By focusing on first-time admissions, we aimed to eliminate potential confounding factors associated with multiple admissions, such as varying treatment responses, changes in health conditions, or differences in care practices across different ICU stays. This approach helps to ensure that the predictions made by our model are based on the initial severity and characteristics of the patients’ conditions, rather than being influenced by previous ICU experiences or interventions.

## Data processing

For the dataset used in the research, a total of 1,295 features were initially considered. The input features are shown with **X** and ${𝐗}_{\text{initial}}\in {\mathbb{R}}^{n\times 1295}$ shows the dimension of the input where n is the number of rows in the dataset. We then followed the Eq 1 to eliminate features containing more than 50% NaN values and using expert opinion to reduce the number of features to 144 that might be related to the target variable. For the retained features, we imputed missing values with the median value. Additionally, we used Eq 2 to normalize the numerical values to standardize the scales and improve convergence.

$${𝐗}_{\text{filtered}}=\{{𝐱}_{i}\in {𝐗}_{\text{initial}}|\text{NaN}({𝐱}_{i})\leq 0.5\times n\}$$ (1)

$${𝐗}_{\text{scaled}}=2\times \frac{{𝐗}_{\text{filtered}}-\min({𝐗}_{\text{filtered}})}{\max({𝐗}_{\text{filtered}})-\min({𝐗}_{\text{filtered}})}-1$$ (2)

## Feature selection

We used XGBoost and LASSO, along with expert opinion, to select key predictors for subsequent analysis. Both XGBoost and LASSO provided feature importance rankings, from which we selected the top contributors. At a certain point in the ranking, there was a noticeable gap in feature importance, and we excluded features below that threshold to ensure only the most impactful variables were retained. The top features identified by both methods were largely similar, and with the input of expert opinion, we further filtered these features down to 30, removing those that were not considered useful for this specific task. In other words, we further validated our selection, we incorporated expert clinical judgment to ensure that the retained features were not only statistically significant but also clinically relevant for predicting ICU stroke mortality. The final 30 features represent a balance between model interpretability, predictive performance, and clinical significance, ensuring that our model remains both robust and generalizable. All physiological test indicators and disease diagnoses were referenced using ICD-9 codes. Table 1 presents the proposed 30 features, including:

**Table 1:** Feature list after applying the XGBoost feature important techniques, expert opinion, and literature review.

No	Feature	No	Feature
1	GCS - Eye Opening	16	HCO3 (serum)
2	O2 Flow (L/min)	17	Chloride (serum)
3	GCS - Verbal Response	18	TCO2 (calc) Arterial
4	GCS - Motor Response	19	Creatinine
5	Intravenous / IV access prior to admission	20	O2 saturation pulseoxymetry (%)
6	Ventilator Type	21	Base Excess
7	Anion Gap	22	BUN
8	Insulin pump	23	Self ADL
9	Arterial CO2 Pressure (mmHg)	24	RDW
10	Respiratory Rate (Total) (insp/min)	25	Respiratory Rate (spontaneous) (insp/min)
11	Braden Nutrition	26	Red Blood Cells
12	O2 Saturation Pulseoxymetry Alarm - High	27	INR(PT)
13	ST Segment Monitoring On	28	Braden Friction/Shear
14	Braden Mobility	29	Daily Weight (kg)
15	marital_status	30	Alarms On

(I) GCS - Eye Opening: The patient’s level of consciousness based on their response to stimuli. (II) O2 Flow (L/min): The rate at which oxygen is administered to the patient. (III) GCS - Verbal Response: The patient’s level of consciousness based on their verbal response to stimuli. (IV) GCS - Motor Response: The patient’s level of consciousness based on their motor response to stimuli. (V) Intravenous / IV Access Prior to Admission: Indicates whether the patient had intravenous access established before ICU admission. (VI) Ventilator Type: Specifies the type of ventilator used for respiratory support. (VII) Anion Gap: The difference between measured cations and anions in the blood. (VIII) Insulin Pump: Indicates whether the patient is using an insulin pump for administering insulin. (IX) Arterial CO2 Pressure (mmHg): The partial pressure of carbon dioxide in arterial blood. (X) Respiratory Rate (Total) (insp/min): The total respiratory rate, measured in breaths per minute. (XI) Braden Nutrition: Assessing a patient’s risk for pressure ulcers related to nutrition. (XII) O2 Saturation Pulseoxymetry Alarm - High (%): The high alarm threshold for oxygen saturation as measured by pulse oximetry. (XIII) ST Segment Monitoring On: Indicates whether ST segment monitoring is activated. (XIV) Braden Mobility: Assessing a patient’s risk for pressure ulcers related to mobility. (XV) Marital Status: The patient’s marital status. (XVI) HCO3 (serum): The concentration of bicarbonate ions in the blood serum. (XVII) Chloride (serum): The concentration of chloride ions in the blood serum. (XVIII) TCO2 (calc) Arterial: The calculated total carbon dioxide content in arterial blood. (XIX) Creatinine: The concentration of creatinine in the blood. (XX) O2 Saturation Pulseoxymetry (%): Oxygen saturation as measured by pulse oximetry. (XXI) Base Excess: The amount of excess or deficit of bases (bicarbonate) in the blood. (XXII) BUN: Blood urea nitrogen. (XXIII) Self ADL: Self-assessed activities of daily living. (XXIV) RDW: Red blood cell distribution width. (XXV) Respiratory Rate (spontaneous) (insp/min): The respiratory rate during spontaneous breathing. (XXVI) Red Blood Cells: The concentration of red blood cells in the blood. (XXVII) INR (PT): International normalized ratio. (XXVIII) Braden Friction/Shear: Assessing a patient’s risk for pressure ulcers related to friction and shear. (XXIX) Daily Weight (kg): The patient’s weight measured daily. (XXX) Alarms On: Indicates whether alarms are activated.

In our research, we applied two models, XGBoost and LASSO, for feature selection, each offering unique benefits. XGBoost is a scalable tree boosting system that excels in achieving high predictive accuracy across various domains, making it a popular choice in machine learning applications [28]. It also includes regularization parameters that help prevent overfitting while capturing complex relationships in the data. Furthermore, XGBoost’s advanced feature selection capabilities enable the identification of the most relevant predictors while minimizing noise, thereby enhancing model interpretability and generalization performance. Despite its widespread adoption, XGBoost’s complex ensemble of decision trees can pose challenges in model interpretation and fine-tuning. The parameters of the used XGBoost are summarized in the Table 2.

**Table 2:** XGBoost model parameters used in this paper and their values.

Parameter	Value
n_estimators	150
base_score	0.5
learning_rate	0.1
max_depth	5
min_child_weight	1
gamma	0
subsample	1
colsample_bytree	1
colsample_bylevel	1
colsample_bynode	1
reg_alpha	0
reg_lambda	1
scale_pos_weight	1
max_delta_step	0

LASSO, a widely used regression technique, is renowned for its ability to perform feature selection and enhance model interpretability [29]. By shrinking regression coefficients towards zero, LASSO encourages sparsity in the model, effectively identifying the most influential predictors [30]. However, LASSO’s variable selection may be biased towards those with higher coefficients, potentially overlooking important but smaller effects [31]. Considering the strengths and weaknesses of each model, we integrated the features identified by both models into the training of our predictive model. The LASSO parameters are shown in Table 3. We determined the ultimate feature selection model based on the accuracy, precision, sensitivity, F1-score, and specificity of the parameters obtained.

**Table 3:** LASSO model parameters used in this paper and their values.

Parameter	Value
alpha	0.005
max_iter	900
tol	0.0001
selection	cyclic

## Modeling

The dataset was imbalanced, with a survival-to-death ratio of 4:1 (1935:505). To address this issue, we implemented the Synthetic Minority Over-Sampling Technique (SMOTE) [32]. Additionally, the train-test split method was used to divide the dataset into training, testing, and validation sets(70/15/15). We developed a novel deep learning neural network to predict mortality in ICU patients with ischemic stroke. For comparison, we established four baseline machine learning models: Random Forest, Logistic Regression, XGBoost, and LightGBM [28, 33–35]. To ensure the robustness and reliability of our predictive models, we implemented five-fold cross-validation to minimize the impact of a single dataset split and provide a comprehensive evaluation of the models’ generalizability and stability."

The choice of a deep learning model over traditional machine learning models was motivated by the need to handle the complex, high-dimensional nature of ICU patient data. Deep learning models are particularly well-suited for capturing non-linear relationships and interactions among multiple features, which are common in healthcare data. Compared to other models, such as Random Forest, Logistic Regression, XGBoost, and LightGBM, deep learning can better learn from the rich, high-dimensional data. Additionally, our deep learning model showed superior performance in preliminary tests, achieving higher AUROC and specificity. This indicates a better ability to reduce false positives and accurately predict patient outcomes, which is crucial in critical care settings. Therefore, we selected deep learning as the primary model for its potential to provide more precise and reliable mortality predictions in ICU stroke patients.

**Fig 3:**
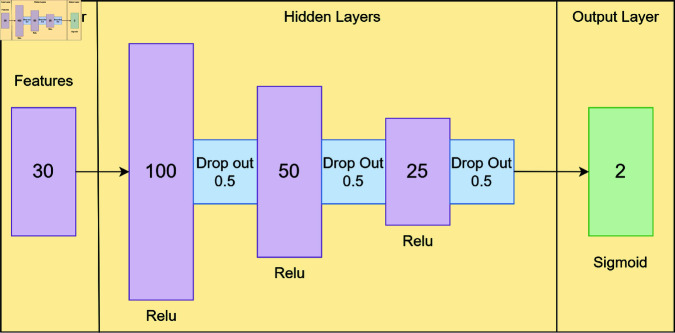
Model architecture. This figure illustrates the neural network structure used, consisting of three hidden layers with 100, 50, and 25 neurons, respectively, each incorporating a dropout rate of 0.5. The model was trained using 30 input features.

Fig 3 illustrates the architecture of our deep learning neural network (NN) model. This model consists of a fully connected NN with an initial layer of 30 dimensions, followed by a batch normalization (BN) layer for input normalization, enhancing the model’s stability [36]. The batch normalization process is defined in Eq 3, here $\mu_{b}$ is the mean value of the batch and $\sigma_{b}$ is the standard deviation of batch, and the ϵ is a small constant to avoid division by zero. The model includes three hidden layers, each employing the rectified linear unit (ReLU) activation function which is defined by Eq 4. Dropout layers were utilized between these hidden layers to mitigate overfitting. The number of neurons decreases from 100 in the first hidden layer to 25 in the third hidden layer. The output layer contains a single neuron, using the sigmoid activation function given in Eq 5 for binary classification, producing output probabilities ranging from 0 to 1. The model was trained using the SGD optimizer, with binary_crossentropy as the loss function and AUROC as the evaluation metric. The training process was ran for 100 epochs with a batch size of 32. This series of operations enhances the model’s ability to distinguish between positive and negative cases. To further clarify the architectural flow, the input layer processes 30 selected features, which are then normalized to stabilize training dynamics. Each hidden layer successively extracts higher-order representations, with neuron counts decreasing (100, 50, and 25) to enforce progressive feature refinement. The ReLU activation at each hidden layer allows efficient learning of non-linear relationships, while dropout layers mitigate overfitting by preventing co-adaptation of neurons. The final output neuron, activated via the sigmoid function, produces a probability score between 0 and 1, determining patient mortality risk. This structured design ensures that the network efficiently captures the intricate dependencies within ICU data while maintaining generalizability.

$${\hat{x}}_{i}=\frac{x_{i}-\mu_{B}}{\sqrt{\sigma_{B}^{2}+\epsilon }}$$ (3)

ReLU(x)=max(0,x) (4)

$$\sigma (x)=\frac{1}{1+e^{-x}}$$ (5)

The best model was selected based on its performance on the validation set. Additionally, we calculated accuracy, precision, sensitivity, F1-score, and specificity to evaluate our models’ performance.

## Ablation process

To assess whether the 30 selected features would adversely affect model performance, we gradually eliminated variables that negatively impacted the model. We evaluated performance on the validation set by calculating the 95% CI of the AUROC. We sequentially deleted one variable at a time, repeating the process until further deletions no longer improved performance. This method filters out non-contributing variables, thereby enhancing model accuracy. After this process, we found all 30 features positively influenced performance, so we decided to retain all features. This algorithm is summarized in Algorithm 1.

## Results

### Cohort comparison

We extracted data for 3,646 ICU patients from the MIMIC-IV database for the development of our model. The cohort was then randomly divided into three subsets: 2,440 patients were allocated to the training set, 682 patients to the test set, and 524 patients to the validation set. The training and validation sets were used to train the models, and the model that achieved the highest AUROC value was selected as the optimal predictive model for further evaluation on the test set. Table 4 provides a comprehensive comparison of demographic and clinical characteristics between the training cohort (N=2440) and the validation cohort (N=524).

**Algorithm 1. Feature selection using AUROC evaluation.**

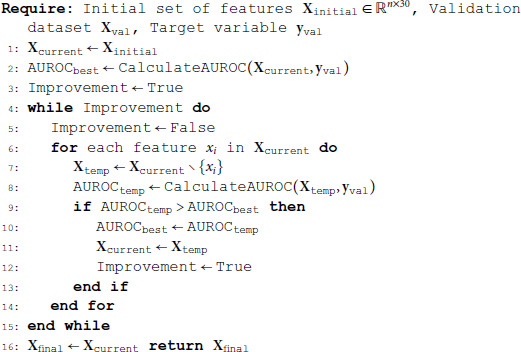



**Table 4:** Comparison of train and validation cohorts. Values from row 9 to row 20 are presented as mean (standard deviation). Some patients’ race information is unknown.

No	Feature	Train Cohort (N=2440)	Validation Cohort (N=524)	P-value
1	Gender [M/F]	[1293/1147]	[273/251]	0.788
2	Target [Survive/Death]	[1935/505]	[421/103]	1.00
3	Race - White	1690 (54.12%)	369 (55.10%)	0.789
4	Race - African American	193 (6.17%)	40 (5.98%)	0.789
5	Race - Hispanic/Latino	92 (2.94%)	12 (1.79%)	0.789
6	Race - Asian	64 (2.05%)	14 (2.09%)	0.789
7	Race - American Indian/Alaska Native	7 (0.22%)	2 (0.30%)	0.789
8	Race - Other	174 (5.57%)	36 (5.61%)	0.789
9	Age	68.01 (15.30)	68.93 (15.43)	0.625
10	GCS - Eye Opening	2.92 (1.13)	2.91 (1.15)	0.399
11	O2 Flow (L/min)	5.59 (5.73)	5.37 (4.47)	0.117
12	GCS - Verbal Response	3.05 (1.74)	3.10 (1.75)	0.055
13	GCS - Motor Response	4.99 (1.48)	4.96 (1.51)	0.070
14	Intravenous	0.55 (0.50)	0.51 (0.50)	0.648
15	Ventilator Type	1.02 (0.23)	1.00 (0.56)	0.590
16	Anion Gap	14.24 (3.41)	14.24 (3.62)	0.999
17	Insulin Pump	0.00 (0.06)	0.00 (0.00)	0.605
18	Arterial CO2 Pressure (mmHg)	38.74 (7.42)	39.53 (9.00)	0.410
19	Respiratory Rate (insp/min)	18.95 (13.24)	18.38 (4.44)	0.101
20	Braden Nutrition	2.38 (0.60)	2.38 (0.59)	0.06

Key demographics such as age, gender, and race exhibit similar distributions across both cohorts, suggesting consistency and the potential for generalizability of the findings. Clinical parameters, including various GCS scores, oxygen flow rates, and other medical metrics, are compared with their mean values and standard deviations for each group. All p-values are greater than 0.05, indicating no statistically significant differences in these parameters between the cohorts, reinforcing the validation cohort’s reliability as a representative sample for further research analysis or model validation.

### Ablation study on variable

**Fig 4:**
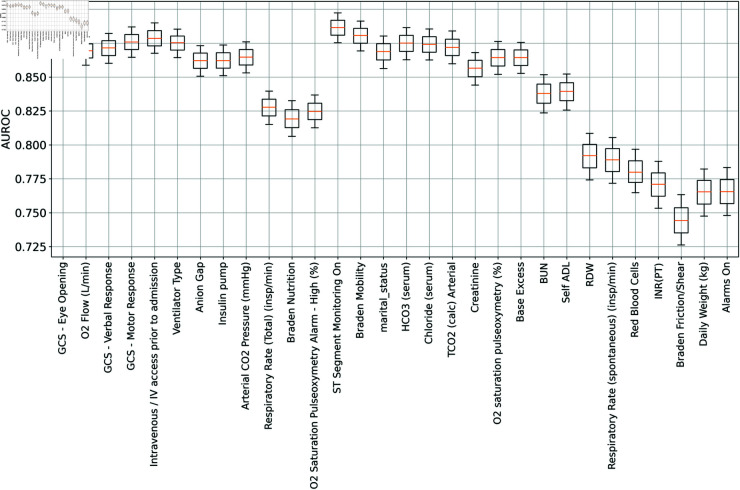
Ablation study. This figure presents the ablation study conducted for this paper. The upper and lower parts of each box plot represent the high and low ranges of the confidence interval, respectively, while the middle point indicates the AUROC.

The ablation study shown in Fig 4 demonstrates that removing any feature negatively impacts the model’s AUROC. None of the values in the figure surpass the original model, which includes all 30 features, and has an AUROC of 0.89.

This superior performance indicates that the baseline configuration already optimally captures the necessary predictive elements. The graphical results from the study show that subsequent ablations, which involve the systematic removal of features such as GCS Eye Opening, GCS Verbal Response, and other clinical variables, do not lead to an increase in AUROC values. In fact, in each instance where a feature is removed, the AUROC tends to decrease or remain unchanged compared to the baseline. This finding underscores that the current feature set within the baseline model is integral to its predictive success. Any removal of these features would not contribute positively to the model’s performance; therefore, maintaining the existing feature composition is advisable. These results validate the robustness of the baseline model and suggest that the included features collectively enhance the model’s ability to accurately predict outcomes, negating the necessity for further adjustments or simplifications in the feature set. This stability in model performance with the existing features supports their continued use without modification for optimal results.

### Evaluation results

**Fig 5:**
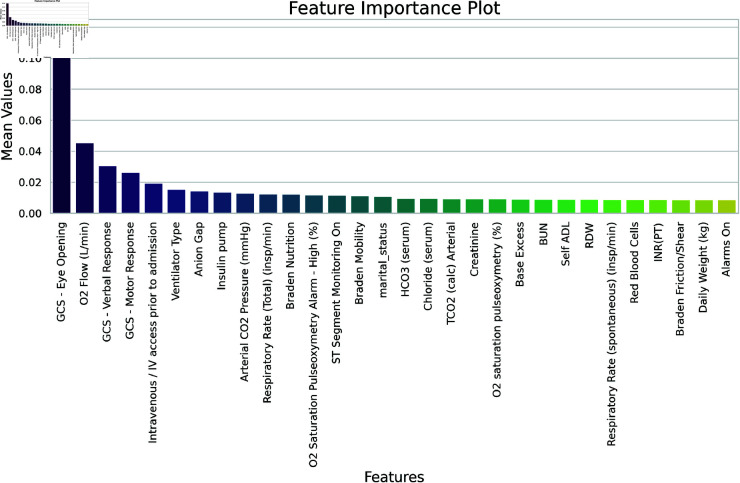
Feature importance. Feature importance and ranking based on XGBoost feature extractor.

Fig 5 shows the importance of each feature as results of XGBoost algorithm. Table 5 summarizes the performance criteria of various machine learning models designed to predict patient mortality, highlighting how each model excels or lags in specific criteria. The LASSO-RF model demonstrates exemplary sensitivity, making it highly effective at identifying patients at high risk of mortality. In contrast, the XGB-LR model boasts the highest precision, indicating its accuracy in confirming cases when a positive result is predicted. Meanwhile, the XGB-RF model balances both precision and sensitivity effectively, achieving the highest F1-score among all models. Notably, the XGB-DL model scores highest in specificity, which is crucial for reducing false positive rates. Each model presents a trade-off between these metrics, reflecting their suitability for different clinical scenarios depending on the desired outcome–whether it’s avoiding false negatives or false positives.

**Table 5:** Accuracy, precision, sensitivity, F1-score, and specificity for different classifiers using XGBoost and LASSO as feature extractors.

	Accuracy	Precision	Sensitivity	F1-Score	Specificity
XGB-RF	0.866	0.865	0.989	0.923*	0.334
XGB-LR	0.783	0.954*	0.769	0.851	0.841
XGB-XGB	0.856	0.870	0.967	0.916	0.373
XGB-LightGBM	0.844	0.858	0.967	0.909	0.308
**XGB-DL**	0.853	0.939	0.836	0.884	0.864*
LASSO-RF	0.876	0.872	0.993*	0.928	0.370
LASSO-LR	0.796	0.942	0.799	0.865	0.787
LASSO-XGB	0.868*	0.874	0.977	0.923*	0.391
LASSO-LightGBM	0.856	0.863	0.978	0.917	0.326
LASSO-DL	0.845	0.918	0.889	0.903	0.655

Selecting the XGB-DL model to improve patient mortality prediction is particularly advantageous due to its high specificity among the evaluated models. Specificity measures the model’s ability to correctly identify true negatives, which, in this context, translates to accurately predicting patients who will not die. This is critical in clinical settings as high specificity minimizes false positives–cases where the model incorrectly predicts death.

However, the model has also produced a relatively low number of false positives (278), which is crucial for improving specificity. This low number of false positives means the model is not overly predicting deaths, helping to prevent unnecessary treatments or interventions for patients inaccurately flagged as high-risk. Moreover, the model has fewer false negatives (833) compared to true positives, indicating a robust balance in sensitivity as well.

Table 6 provides AUROC scores and 95% Confidence Intervals (CI) for a predictive model that assesses patient outcomes every 8 hours across training, validation, and test datasets. In the training set, the model shows exceptional performance with an AUROC of 0.945 and a very tight confidence interval between 0.944 and 0.947, demonstrating consistent accuracy within this dataset. However, a noticeable decline in performance is observed when the model is applied to the validation and test sets, with AUROCs of 0.876 and 0.878, respectively. The slightly broader confidence intervals of 0.865-0.889 for validation and 0.866-0.888 for test indicate more variability in the model’s performance on new, unseen data. This drop suggests that while the model is highly effective with training data, its generalizability is somewhat limited, possibly due to overfitting. This observation underscores the necessity for additional model tuning or adjustments in model complexity to enhance its applicability across diverse datasets.

**Table 6:** AUROC and 95% confidence interval for train, validation, and test sets.

Set	AUROC	95% CI
Train set	0.945	[0.944 - 0.947]
Validation set	0.876	[0.865 - 0.889]
Test set	0.878	[0.866 - 0.888]

In parallel, Table 7 details the performance of the same predictive model over the initial four days, highlighting a progressive improvement in its ability to accurately forecast patient outcomes. Starting with an AUROC of 0.865 on the first day, the score steadily increases to 0.903 by the fourth day. The accompanying 95% CIs for each day’s AUROC also tighten significantly by the fourth day, ranging from 0.868 to 0.936, which boosts confidence in the model’s predictions as more data is analyzed over time. In the last column of this table, our results are compared with the previous study which shows a huge improvement (11-15%) in AUROC. In the previous best model, the AUROC does not improve over time necessarily, leading to a weak model for predicting mortality over time. In contrast, our model demonstrates a significant progressive improvement, making it a valuable tool for mortality prediction over time, which is of utmost importance. Note that previous study has not reported CI for their AUROC values.

**Table 7:** AUROC comparison of XGB-DL with previous best study, including 95% confidence interval for our model.

Day	Proposed Model AUROC	95% CI	Best Model AUROC [20]
Day 1	0.865	[0.821 - 0.905]	0.742
Day 2	0.882	[0.844 - 0.920]	0.776
Day 3	0.882	[0.841 - 0.917]	0.754
Day 4	0.903	[0.868 - 0.936]	0.750

The AUROC metric is particularly crucial in clinical applications because it measures the model’s ability to distinguish between patients at high and low risk of mortality. A higher AUROC indicates better discriminatory performance, which is essential for ICU decision-making. In Table 6, we report AUROC scores across training, validation, and test datasets. The confidence intervals (CIs) provide additional insight into the model’s stability, with narrower CIs in the training set indicating lower variability, while broader CIs in the validation and test sets reflect increased uncertainty when applied to new patient data.

**Fig 6:**
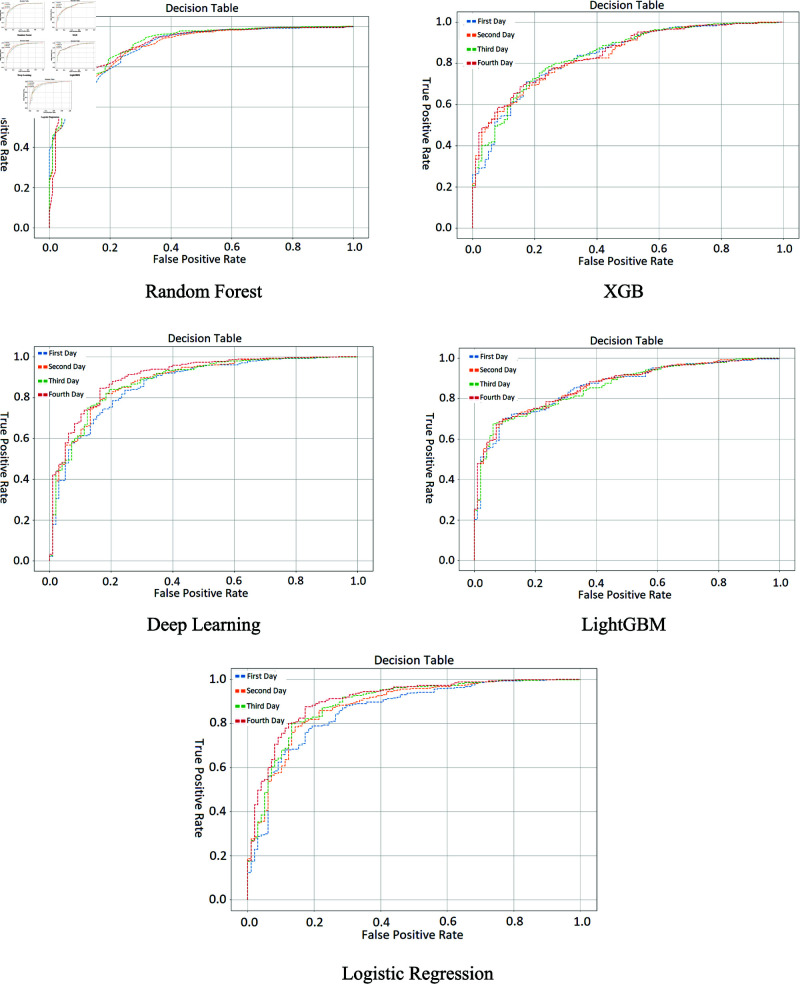
AUC comparison. AUC comparison of different classifiers in four days.

The ROC curve for different methods is plotted in Fig 6. This figure demonstrates that due to our effective feature selection, all our machine learning models outperformed the top study in terms of AUROC.

Deep learning model in this figure, climbing closer to the top-left corner, visually confirms the trend of improvement over days, indicating a significant enhancement in the model’s reliability in predicting patient mortality. This trend underscores the model’s increasing effectiveness at prognosticating outcomes as it processes an expanding dataset across consecutive days. Table 8 Shows the AUROC and 95% confidence interval for different implemented algorithms used in this paper. It is important to note that all of these models outperform the best study. Although Random Forest and Logistic Regression perform well in terms of AUROC, as does Deep Learning, we choose Deep Learning because of its higher specificity.

**Table 8:** AUROC scores with 95% CI from Day 1 to Day 4 for different algorithms.

Day	Random Forest	Logistic Regression	XGBoost	LightGBM	Deep Learning
Day 1	0.8943 [0.8603-0.9255]	0.8604 [0.8132-0.9008]	0.8362 [0.7898-0.8809]	0.8590 [0.8199-0.8959]	0.8657 [0.8214-0.9057]
Day 2	0.8985 [0.8627-0.9263]	0.8793 [0.8369-0.9159]	0.8393 [0.7984-0.8780]	0.8631 [0.8245-0.8972]	0.8827 [0.8448-0.9208]
Day 3	0.9092 [0.8766-0.9370]	0.8910 [0.8509-0.9254]	0.8427 [0.7995-0.8837]	0.8584 [0.8251-0.8930]	0.8825 [0.8417-0.9180]
Day 4	0.9062 [0.8722-0.9373]	0.9078 [0.8703-0.9388]	0.8458 [0.8044-0.8832]	0.8652 [0.8286-0.8968]	0.9039 [0.8686-0.9362]

### SHAP analysis

**Fig 7:**
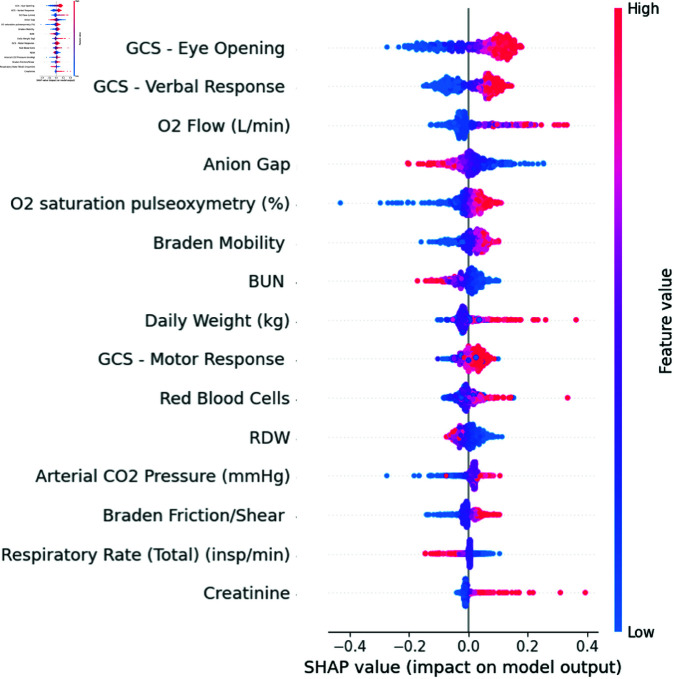
SHAP analysis. SHAP value based on neural network model for the test set.

The SHAP (SHapley Additive exPlanations) analysis graph effectively utilizes machine learning techniques to quantify and visually represent the significance of various clinical parameters in a predictive model. This analysis robustly interprets the impact of individual features on the model’s predictions, enhancing our understanding of the underlying mechanisms driving outcomes [37]. Fig 7 illustrates the influence of the top 15 features on the output of the predictive model, highlighting the importance of each feature in shaping the model’s predictions.

The Glasgow Coma Scale (GCS) scores (Eye Opening, Verbal Response, Motor Response) were among the top contributors to the model’s predictions. Higher GCS scores, reflecting better neurological function, were associated with lower mortality risk, which aligns with clinical practice where patients exhibiting a higher level of consciousness typically have a better prognosis. Oxygen-related variables such as O2 Flow and O2 Saturation Pulseoxymetry displayed both positive and negative SHAP values. These parameters are critical in managing respiratory support, and their variation reflects different clinical scenarios, such as acute respiratory distress or recovery phases. For instance, higher oxygen flow may indicate severe respiratory compromise, while stable oxygen saturation levels are indicative of controlled respiratory function. Laboratory parameters like Anion Gap and BUN (Blood Urea Nitrogen) were significant in assessing metabolic and renal function. An elevated Anion Gap often signals metabolic acidosis, a common complication in critically ill patients, and correlates with higher mortality risk. Similarly, increased BUN levels are typically seen in patients with renal dysfunction or dehydration, both of which are poor prognostic factors in the ICU setting.

Physiological measures like Creatinine and Daily Weight are directly linked to renal function and fluid balance. Elevated Creatinine levels suggest impaired kidney function, while abnormal weight fluctuations may reflect fluid imbalances, both of which contribute to the patient’s overall mortality risk. Respiratory Rate and Arterial CO2 Pressure add further granularity to the model’s understanding of respiratory health. Abnormalities in these values are associated with respiratory failure or inadequate ventilation, which are critical concerns for ICU patients. The SHAP analysis effectively highlights the importance of these variables in predicting patient outcomes, providing actionable insights for clinical decision-making.

## Discussion

### Existing model compilation summary

This study successfully developed a deep learning approach that significantly enhances the prediction of mortality among ICU patients suffering from ischemic stroke. Compared to best-existing literature [20], all of our models improved the AUROC significantly and the baseline model demonstrates 13% improvement on average on AUROC by utilizing a carefully curated set of 30 features, a substantial reduction from [20] which uses 1095 features. we achieved higher accuracy using a model on a dataset with more than 30 times fewer features. This remarkable result underscores the effectiveness of our innovative feature selection techniques and the robustness of our modeling approach. By drastically reducing the feature set, we not only simplified the model but also enhanced its performance and generalization capabilities. Also, this leads to a lot of calculation reduction which makes this model much faster. Additionally, [38] reported an AUROC of 0.700 in the external validation cohort for stroke patients, indicating potential overfitting and limited generalizability, which is notably lower than our AUROC value. [39] employed a Random Forest model for critically ill patients with embolic stroke using the MIMIC-IV dataset, achieving a final AUROC of 0.838, further highlighting the superiority of our approach.

One of the standout features of the XGB-DL model is its specificity, which reaches up to 86.4% in distinguishing true negatives. This aspect is crucial in the clinical environment, where accurate prediction of patient outcomes can significantly influence treatment decisions and resource allocation. Moreover, the model’s AUROC improved progressively from 86.5% (CI 82.1% - 90.5%) to 90.3% (CI 86.8% - 93.6%) over the first four days of patient admission, indicating increasing predictive accuracy that could be pivotal for clinical interventions during critical early stages.

Several studies have demonstrated the potential of machine learning models in improving patient outcomes, especially when integrated with decision-support systems in ICUs. For example, a model similar to ours–as detailed in [40], which analyzed a large dataset of stroke ICU admissions in Brazil to develop machine learning algorithms predicting prolonged hospital stays and short-term mortality–has been shown to enhance resource allocation by alerting healthcare professionals to high-risk patients in a timely manner, thereby enabling earlier interventions. Similarly, [41] utilized machine learning techniques on stroke ICU patients from the Almazov National Research Center, incorporating MRI, ultrasound, and laboratory data to predict mortality outcomes. These models can lead to actionable changes by enabling more personalized treatment plans and adjusting the level of monitoring based on the predicted risk levels. However, these benefits are largely depend on the seamless integration of such models with existing electronic health record (EHR) systems, which remains a challenge in many healthcare settings.

### Study limitations

One limitation of this study is the exclusion of patients with recurrent ICU admissions, which may reduce the model’s ability to capture long-term trends in patient deterioration and recovery. Recurrent admissions often indicate chronic conditions, complications, or treatment failures, which are critical factors in mortality prediction. By excluding these cases, the model primarily reflects first-time ICU stays, which may limit its applicability to patients with complex, long-term medical histories. Future research could incorporate longitudinal patient records to assess how prior ICU stays influence mortality risk, improving the model’s predictive power for patients with recurring critical conditions.

Another key limitation is the reliance on a single dataset, MIMIC-IV, which may introduce biases due to the specific demographic and clinical practices of the institutions contributing to this database. Since ICU protocols, patient populations, and healthcare resources vary across hospitals and geographic regions, a model trained solely on MIMIC-IV may not generalize well to other clinical settings. External validation using datasets from multiple healthcare systems, including non-U.S. hospitals, would help assess the model’s robustness and adaptability to different patient populations.

Additionally, while median imputation was used to handle missing data, this approach assumes that missing values are randomly distributed, which may not always be the case in clinical datasets. Certain missing values might be systematically related to patient severity or specific treatment pathways, potentially biasing the model’s predictions. More advanced imputation techniques, such as multiple imputation by chained equations (MICE), deep generative models, or domain-specific imputation strategies, could enhance data completeness and preserve underlying relationships within the dataset. Future studies should explore these methods to reduce information loss and improve the model’s reliability in real-world clinical applications.

## Conclusion

This research significantly advances predictive modeling of mortality in ischemic stroke patients within ICU settings. Although Random Forest and Logistic Regression also performed well in terms of AUROC, we chose Deep Learning because of its higher specificity. The XGB-DL model, with its high specificity and improved predictive accuracy over time, promises to be a valuable tool for clinicians, enhancing patient outcomes and optimizing ICU resource utilization. Notably, our approach achieved an impressive 13% increase in AUROC on average, while utilizing 30 times fewer features, demonstrating the model’s efficiency and effectiveness.

Future studies should aim to validate this model across varied healthcare databases to ascertain its effectiveness and adaptability across different patient demographics and treatment protocols. Additionally, exploring the integration of this predictive model into clinical practice could provide insights into operational challenges and benefits, paving the way for broader adoption and potentially transforming ICU patient care management.
